# CRISPR/Cas9-Mediated Knockout of the Chorion Gene *s36* Impairs Ovarian Development and Abolishes Egg Hatching in the Melon Fly *Zeugodacus cucurbitae*

**DOI:** 10.3390/insects17101024

**Published:** 2026-10-04

**Authors:** Lina Sun, Xinlin Fan, Ting Chen, Qingxing Shi, Guojun Qi, Bin Tang, Jie Chen

**Affiliations:** 1 Institute of Plant Protection, Guangdong Academy of Agricultural Sciences, Key Laboratory of Green Prevention and Control on Fruits and Vegetables in South China Ministry of Agriculture and Rural Affairs, Guangdong Provincial Key Laboratory of High Technology for Plant Protection, Guangzhou 510640, China; 2 School of Life Sciences, Hangzhou Normal University, Hangzhou 311121, China

**Keywords:** *Zeugodacus cucurbitae*, genome editing, oogenesis, eggshell permeability, vitellogenin, female sterility, genetic pest control

## Abstract

The melon fly, *Zeugodacus cucurbitae* (Coquillett), is a globally invasive pest of cucurbit crops whose concealed larval feeding limits chemical control and has driven interest in reproduction-based management. The chorion gene *s36* encodes an eggshell protein conserved among dipterans. However, whether *s36* is required for female reproduction in *Z. cucurbitae* remains unknown. We generated a homozygous *s36* mutant line by CRISPR/Cas9 and characterized its reproductive phenotype. Disruption of *s36* strongly reduced transcript abundance and truncated the predicted protein. Mutant females showed elevated mortality after sexual maturation, fecundity was approximately halved, and none of the eggs laid hatched. Ovaries were markedly reduced in size and were scored as abnormal in every mutant female examined, and eggs were smaller, dull and shriveled rapidly in air, indicating compromised chorion integrity. Both vitellogenin genes were down-regulated in the ovary. Whole-body transcriptomics identified 179 differentially expressed genes enriched in lipid and hormone-related metabolism. Tissue-resolved RT-qPCR showed vitellogenin and *yellow-g2* transcripts elevated in the fat body yet depleted in the ovary; whether this pattern reflects impaired delivery of yolk precursors to the oocyte or the secondary loss of the follicular tissue in which these genes are normally expressed remains to be determined. These findings identify *s36* as required for normal ovarian maturation and egg viability in *Z. cucurbitae*, and as a candidate target for genetic control of this pest.

## Simple Summary

The chorion gene s36 encodes an eggshell protein conserved among dipterans, but whether it is required for female reproduction in the melon fly, Zeugodacus cucurbitae, was unknown. We generated a homozygous s36 mutant line using CRISPR/Cas9. Mutant females showed elevated mortality after sexual maturation, approximately halved fecundity, and no egg hatching at all; ovaries were markedly reduced in size and eggs shriveled rapidly in air, indicating compromised chorion integrity. Transcriptomics and tissue-resolved RT-qPCR revealed altered lipid and hormone-related metabolism and tissue-specific changes in vitellogenin and yellow-g2 expression. These findings identify s36 as required for normal ovarian maturation and egg viability and as a candidate target for genetic control of this pest.

## 1. Introduction

Insect pests of the family Tephritidae cause severe losses to fruit and vegetable production worldwide and are among the most frequently intercepted quarantine organisms in international trade [1]. The melon fly, *Zeugodacus cucurbitae* (Coquillett), is one of the most destructive members of this family, attacking more than one hundred host plants with a strong preference for cucurbits such as bitter gourd, cucumber and ridge gourd [2]. Females oviposit into developing fruit and the larvae feed internally, so that infested fruit rots and drops before harvest; in heavily infested plantings the marketable yield can be lost almost entirely [3]. Because the immature stages are concealed within the host tissue, foliar insecticides reach them poorly, and repeated applications targeted at adults have selected for resistance in field populations of this species [4]. Broad-spectrum insecticide use also carries well-documented non-target costs [5,6]. Current management therefore combines physical exclusion, host-derived attractants and biological control agents [3,7,8,9], and the chemo-sterilization has been explored as a means of suppressing reproduction directly [10]. The efficacy of each of these measures is nonetheless sensitive to environmental conditions, which are themselves shifting [11], and alternative approaches that are effective, species-specific and compatible with integrated management remain needed.

Interfering with female reproduction is an attractive route to such alternatives, because a female that lays fewer eggs, or eggs that cannot develop, contributes nothing to the next generation [12]. Insect oogenesis proceeds through the coordinated proliferation of germline and somatic follicle cells, the massive uptake of yolk precursors, and the deposition of an eggshell around the mature oocyte [13,14]. Germline stem cell maintenance and follicle cell specification are governed by conserved signaling pathways, including Hedgehog, JAK/STAT and Wnt, together with transcriptional regulators acting within the germarium [15,16,17,18,19]. Vitellogenins are subsequently synthesized extra-ovarially, taken up by developing oocytes through the vitellogenin receptor, and stored as the principal nutrient reserve of the embryo [20,21,22,23], a process placed under endocrine control by juvenile hormone and ecdysteroid signaling [24,25,26]. Genes acting at each of these steps are required for fertility in tephritids and other pests, and their disruption by RNA interference or genome editing produces measurable reproductive deficits [27,28,29,30,31,32]. RNA interference has been the principal tool for such functional tests since its discovery [33,34] and is now being translated towards field application [35], although its efficiency varies widely among species and is constrained in *Z. cucurbitae* by extracellular dsRNA degradation [36,37]. RNA interference has moved closer to field use over the past few years. In tephritids, the main obstacle is degradation of ingested dsRNA by gut nucleases, and knocking down these nucleases markedly improves silencing efficiency in *Ceratitis capitata* and *Bactrocera tryoni* [38]. Feeding adults dsRNA against gut nucleases together with dsRNA against a lethal target enhances the response further in *C. capitata* [39]. Products have also reached the market: ledprona, a sprayable dsRNA biopesticide targeting a proteasome subunit of the Colorado potato beetle, was the first such product to be registered [40], and field trials have since been authorized in Europe. Stable genetic knockout therefore offers a more decisive test of gene function in this pest.

The eggshell is the final and outermost product of oogenesis. In dipterans it is secreted by the follicular epithelium during the terminal stages of oocyte maturation and consists largely of chorion proteins, which polymerize into a multilayered structure that protects the embryo against mechanical damage and desiccation while permitting gas exchange and sperm entry [41]. Structural proteins secreted around the developing oocyte serve comparable protective roles in other arthropods [42], and mucin-type components deposited at the egg surface are required for early embryogenesis in insects [43]. Among the chorion components, *s36* is one of the earliest chorion genes to be expressed and has been characterized in both *Drosophila melanogaster* and the Mediterranean fruit fly *Ceratitis capitata*, in which its coding and regulatory sequences are conserved [44]. Targeted downregulation of *s36* in *D. melanogaster* compromises chorion biogenesis and architecture [45] and renders ovarian follicles unable to support sperm entry [46], establishing *s36* as a structural determinant of eggshell function. Evidence in *D. melanogaster* rests on RNA interference rather than on a null allele, however, and whether *s36* is likewise required for female reproduction in a pest, and whether its loss affects oogenesis upstream of eggshell assembly, remains largely unknown. We hypothesized that complete loss of *s36* would compromise eggshell competence and, because chorion deposition is the terminal step of a tightly coupled maturation program, would also be reflected in the ovary itself. Beyond its structural role, the chorion is the principal barrier limiting entry of exogenous molecules into the insect egg, so genes governing chorion assembly bear directly on delivery-based control strategies. Eggs of most Diptera are laid inside host tissue and are poorly accessible to sprayed formulations, whereas eggs of many Lepidoptera and Coleoptera are exposed and can be treated directly [47,48]. Characterizing *s36* by complete loss of function therefore addresses both how the eggshell is built and whether its composition can be manipulated to change embryonic permeability.

CRISPR/Cas9 mutagenesis now provides direct genetic evidence for gene function in non-model insects, including tephritids [49,50,51], and has been applied both to dissect physiological traits [52] and to validate reproduction-related targets and build genetic control systems in several pest species [53,54,55,56]. In this study, we generated a homozygous *s36* mutant line of *Z. cucurbitae* by CRISPR/Cas9 mutagenesis and examined its survival, fecundity, egg hatching, ovarian and egg morphology, vitellogenin expression and the whole-body transcriptome. We show that loss of *s36* impairs ovarian development, compromises eggshell integrity and abolishes egg hatching, identifying *s36* as a candidate target for reproduction-based control of this pest.

## 2. Materials and Methods

### 2.1. Insect Rearing

The *Z. cucurbitae* colony was maintained by the Institute of Plant Protection, Guangdong Academy of Agricultural Sciences, in a climate-controlled room at 26 ± 1 °C, 60–70% relative humidity and a 14 h:10 h photoperiod. Adults were held in 30 × 30 × 30 cm gauze cages and fed a mixture of yeast extract and glucose (1:3, *w*/*w*); after sexual maturation the diet was changed to yeast extract and glucose (1:1, *w*/*w*). Water was supplied continuously. Fresh courgetti slices were provided as an oviposition substrate, larvae were reared on the same host tissue, and mature larvae were transferred to moist sand for pupation. Pupae were sieved out after 3 d and transferred to clean cages for adult emergence.

### 2.2. sgRNA Design and In Vitro Synthesis

The exon–intron structure of *s36* was determined with SnapGene, and target sites were placed preferentially in long exons to avoid the reduced editing efficiency and splicing ambiguity associated with intronic targets. Candidate sequences were submitted to CRISPOR (http://crispor.org) and scored for specificity, position, predicted efficiency and off-target probability against the *Z. cucurbitae* reference genome (NCBI GCF_028554725.1); two sgRNA target sites were retained for *s36* (NCBI 105210599), and the site 5′-GCCAGCCTGTTGAGCACCAC**CGG**-3′ (positions 383–403 bp; PAM in bold) was used to establish the mutant line described here.

Transcription templates were prepared by overlap PCR using a forward primer carrying a T7 promoter followed by the 20 nt target sequence and a universal reverse primer encoding the full tracrRNA scaffold (Table S1). Reactions contained 0.5 µL template, 1 µL of each primer, 12.5 µL 2× Taq Plus Master Mix and ddH_2_O to 25 µL, and were run at 98 °C for 2 min; 40 cycles of 98 °C for 10 s, 55 °C for 10 s and 72 °C for 45 s; and 72 °C for 10 min. sgRNAs were transcribed with the T7 RiboMAX Express RNAi System (Promega, Madison, WI, USA) at 37 °C for 2 h, treated with RQ1 RNase-free DNase, precipitated with sodium acetate and ethanol, washed twice with 70% ethanol and dissolved in DEPC-treated water. Integrity was checked on a 1% agarose gel and concentration was determined spectrophotometrically.

### 2.3. Embryonic Microinjection

sgRNA was mixed with TrueCut Cas9 Protein v2 (Thermo Fisher Scientific, Waltham, MA, USA) in nuclease-free water to final concentrations of 500 ng/μL sgRNA and 100 ng/μL Cas9, and held on ice for at least 40 min before use. Injections were performed using borosilicate glass capillaries (IB100-4, WPI, Sarasota, FL, USA) pulled on a vertical pipette puller (PC-100, NARISHIGE) (heating temperature 60 °C, with two light and two heavy weights, 250 g in total; capillaries were removed after the heating filament had faded from red and cooled naturally) mounted on an oil-pressure micromanipulation pump and micromanipulator (IM-21, NARISHIGE). Fresh cucumber slices were placed in cages of sexually mature adults and inspected every 30 min; newly laid eggs were collected with a fine brush, transferred to double-sided adhesive tape on a glass slide, dechorionated manually, aligned, briefly air-dried and covered with a 1:1 mixture of halocarbon oil 27 and halocarbon oil 700 to prevent desiccation. The injection of a batch was completed within 2 h. More than 500 embryos were injected, of which 152 hatched in a climate-controlled room at 26 ± 1 °C and 60–70% relative humidity.

### 2.4. Screening and Maintenance of Mutant Lines

Injected eggs were incubated until hatching, and larvae were transferred to artificial diet and reared to adulthood. Genomic DNA was extracted from a mesothoracic leg of each adult by grinding the tissue in liquid nitrogen, adding 50 µL of lysis buffer containing proteinase K (AM2546, Invitrogen, Waltham, MA, USA) at a final concentration of 0.4 mg/mL, incubating at 56 °C for 30 min with brief vortex every 10 min, inactivating at 95 °C for 5 min and collecting the supernatant after centrifugation at 13,000 rpm for 2 min. The target region was amplified with the primers listed in Table S1 and sequenced. Individuals whose chromatograms showed superimposed peaks downstream of the cut site were scored as mutants. In total, 35 G_0_ adults and 18 F_1_ adults were screened by sequencing of the target region.

G_0_ mutants were backcrossed to wild-type adults, and F_1_ individuals were genotyped to classify mutation types. Heterozygotes carrying the same mutation were intercrossed to generate F_2_ homozygotes, which were identified by sequencing. Because homozygous mutant females are completely sterile, the line could not be maintained by intercrossing homozygotes; heterozygotes were therefore propagated continuously and homozygotes were selected by genotyping in each generation for phenotypic analysis.

### 2.5. RNA Extraction and RT-qPCR

Total RNA was extracted with TRIzol (Thermo Fisher Scientific, Waltham, MA, USA), and its integrity and concentration were checked by agarose gel electrophoresis and spectrophotometry. First-strand cDNA was synthesized with the PrimeScript RT reagent Kit with gDNA Eraser (TaKaRa, Kusatsu, Japan). RT-qPCR was performed in 20 µL reactions containing 10 µL SYBR Green qPCR Mix (2×), 0.45 µL of each primer and cDNA template, under the following program: 95 °C for 5 min; 40 cycles of 95 °C for 10 s, 60 °C for 20 s and 72 °C for 20 s. Amplification and melting curves were inspected before analysis. *rps3* (NCBI 105210056) was used as the reference gene because it has been validated as a stable reference for RT-qPCR in *Z. cucurbitae* under comparable conditions [57], and relative expression was calculated by the 2^−ΔΔCt^ method [58]. For each group, one healthy sexually mature female was sampled per replicate, with three biological replicates. For the tissue-resolved assay, ovaries and fat body were dissected from sexually mature females in chilled phosphate-buffered saline and processed separately under the same protocol, with three females pooled per replicate and three biological replicates per tissue and genotype. Primers of *s36* (XM_011181658.3), *Vg-1* (XM_011193412.3), *Vg-2* (XM_011193407), and *yellow-g2* (XM_011184526.3) are listed in Table S1.

### 2.6. Adult Mortality

Thirty newly emerged adults of the four genotypes (homozygous mutant females and males, heterozygous females and males) were held under the rearing conditions described above. Thirty *wt* adults, male and female, were used as controls. Dead individuals were recorded daily at 09:00, and cumulative mortality was calculated at 21 d after emergence, with three biological replicates per genotype.

### 2.7. Fecundity and Egg Hatching

Females were reared to sexual maturity (15 d after emergence) and paired with wild-type males at a ratio of 1:3. Beginning 48 h after pairing, fresh cucumber slices were offered daily between 14:00 and 16:00 and removed after 2 h, and eggs were counted under a stereomicroscope. Oviposition was recorded for seven consecutive days. Each group comprised 10 females and three biological replicates. Females in each replicate were housed together in the same cage and their eggs were collected collectively. Eggs were collected and counted per cage, and the number of eggs per female was obtained by dividing the cage total by the number of surviving females. To determine hatching, eggs were transferred to moist cucumber tissue and the number of hatched larvae was counted after a further 48 h.

### 2.8. Ovary and Egg Morphology

Ten sexually mature females per genotype were dissected, and the intact ovary pair was rinsed in PBS, mounted on a glass slide and imaged by three-dimensional reconstruction using an ultra-depth-of-field microscope. Ovary size and the presence of developing oocytes were recorded, and each ovary was scored as normal or abnormal. Eggs laid by each genotype were imaged under the same system. To assess the capacity of the eggshell to retain water, ten freshly laid eggs were placed on a glass slide at room temperature and photographed at 10, 20, 30, 40 and 120 s after exposure to air. The weights of ten eggs were also measured after being left for different periods of time. Three biological replicates were performed.

### 2.9. Transcriptome Sequencing and Analysis

Homozygous mutant and heterozygous females were collected at sexual maturity, with one female per replicate and three biological replicates per genotype. Samples were frozen in liquid nitrogen and sent to Ribobio (Guangzhou, China) for RNA sequencing. The quantity and integrity of RNA yield were assessed by using the K5500 (Kaiao, Beijing, China) and the Agilent 2200 Tape Station (Agilent Technologies, Santa Clara, CA, USA) separately. Briefly, the mRNA was enriched by oligo dT according to the instructions of the NEB Next^®^ Poly(A) mRNA Magnetic Isolation Module (NEB, Ipswich, MA, USA), and it was then fragmented to approximately 200 bp. Subsequently, the RNA fragments were subjected to first strand and second strand cDNA synthesis followed by adaptor ligation and enrichment with a low-cycle according to the instructions of the NEB Next^®^ Ultra™ RNA Library Prep Kit for Illumina. The purified library products were evaluated using the Agilent 2200 Tape Station and Qubit (Thermo Fisher Scientific, Waltham, MA, USA). The libraries were sequenced by Illumina (Illumina, San Diego, CA, USA) with paired-end 150 bp at Ribobio Co., Ltd. (Ribobio, China). The clean reads were obtained after removal of reads containing adapter, ploy-N and low-quality reads from raw data. HISAT2 was used to align the clean reads to the reference genome (GCF_028554725.1) with default parameters. HTSeq was subsequently employed to convert aligned short reads into read counts for each gene model. Principal component analysis was performed on gene expression values to assess the relationship among samples. The statistically significant DE genes were obtained by an adjusted *p*-value threshold of <0.05 and |log2 (fold change)| > 1 using the DEseq/DESeq2/edgeR/DEGseq software. In order to identify the Gene Ontology (GO) annotations and pathways in which differentially expressed genes were enriched, GO term and Kyoto Encyclopedia of Genes and Genomes (KEGG) pathway enrichment analyses were performed using the “cluster Profiler” package in R Bioconductor software. Because the number of DEGs was small, gene set enrichment analysis (GSEA) was additionally performed on the ranked expression of all detected genes against KEGG gene sets. Sets with a nominal *p* < 0.05 are reported together with their normalized enrichment scores and false discovery rate *q* values.

### 2.10. Statistical Analysis

Data are presented as mean ± standard error. Specifically, *s36* transcript abundance, fecundity and the tissue-resolved transcript data were compared by independent-samples *t*-test; cumulative mortality was compared by one-way analysis of variance followed by Duncan’s multiple range test (*p* < 0.05). Egg hatching was not tested statistically because all replicate values of the mutant group were zero. All analyses were performed in IBM SPSS Statistics 21 (IBM, Armonk, NY, USA), and figures were prepared in GraphPad Prism 8.0.

## 3. Results

### 3.1. Generation of a Homozygous s36 Mutant Line

**Figure 1:**
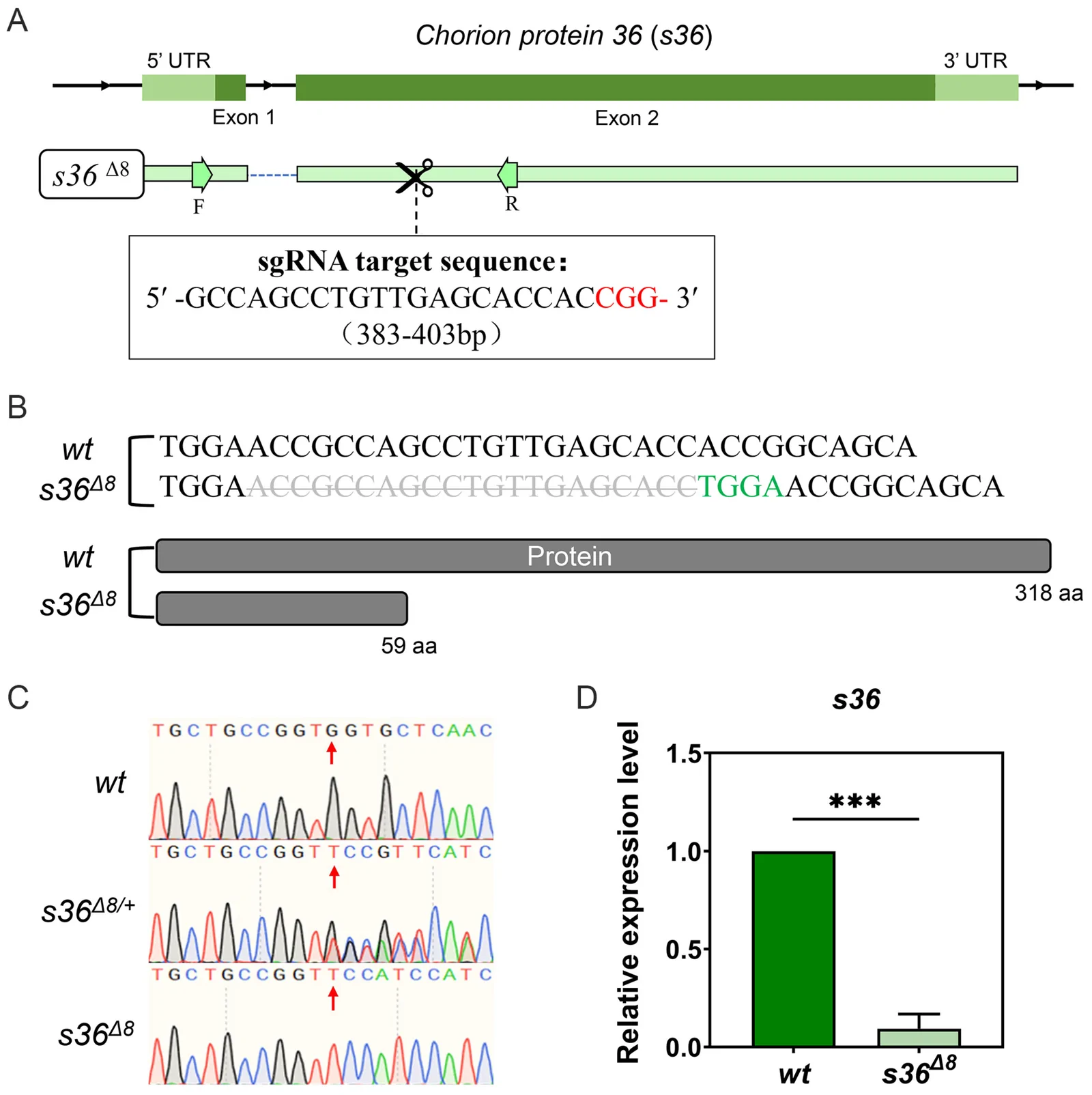
**Generation of the *s36^Δ8^* mutant line of *Z. cucurbitae*.** (**A**) Position of the sgRNA target site within *s36*. The PAM is shown in red and the scissors mark the expected Cas9 cleavage site. (**B**) Alignment of the wild-type and *s36^Δ8^* target regions, with deleted bases struck through and inserted bases in green, and the resulting truncation of the predicted protein from 318 to 59 amino acids. (**C**) Sequencing chromatograms of wild-type (*wt*), heterozygous (*s36^Δ8/+^*) and homozygous (*s36^Δ8^*) individuals. Arrows mark the mutation sites. (**D**) Relative expression of *s36* in wild-type and homozygous mutant females determined by RT-qPCR, with *rps3* as the reference gene and the wild-type level set to 1. Data are shown as mean ± SEM (*n* = 3). *** indicates a significant difference at *p* < 0.001 (Student’s *t*-test).

To obtain direct genetic evidence for the function of *s36*, we targeted the gene by CRISPR/Cas9 mutagenesis using an sgRNA directed against positions 383–403 bp of the coding sequence by evaluating the sgRNA position, cleavage efficiency, and off-target efficiency (Figure 1A). A total of 35 G_0_ adults were obtained, all of which were screened by sequencing of the target region. Of these, 24 G_0_ were identified as edited individuals. The edited G_0_ individuals were backcrossed to *wt* for F_1_ propagation. A total of 18 F_1_ adults were screened by sequencing. All of them were edited individuals carrying the mutation, and no unedited individuals were detected. Sequencing of F_1_ individuals identified six independent mutation types, four of which shifted the reading frame and introduced a premature stop codon, whereas two did not alter the frame (Table S2). The allele designated *s36*^Δ8^, which carries a 21 bp deletion combined with a 4 bp insertion, introduced the earliest premature termination and was therefore selected for functional analysis: the predicted product is truncated from 318 to 59 amino acids (Figure 1B). Homozygous individuals were identified by the recovery of a clean single-peak chromatogram that differed in sequence from the wild-type, in contrast to the superimposed peaks of heterozygotes (Figure 1C). Consistent with the introduction of a premature stop codon, *s36* transcript abundance in homozygous mutant females fell to 9.33% of the wild-type level (*p* < 0.001, Student’s *t*-test; Figure 1D). Transcripts carrying a premature termination codon are recognized and degraded by nonsense-mediated mRNA decay (NMD), a surveillance pathway conserved across eukaryotes that couples translation termination position to transcript stability [59]. Because homozygous mutant females proved to be completely sterile, the line was maintained by intercrossing heterozygotes and selecting homozygotes by genotyping in every generation. These results indicated that a loss-of-function *s36* allele had been successfully established in *Z. cucurbitae*.

### 3.2. Loss of s36 Elevates Mortality and Reduces Fecundity in Females

**Figure 2:**
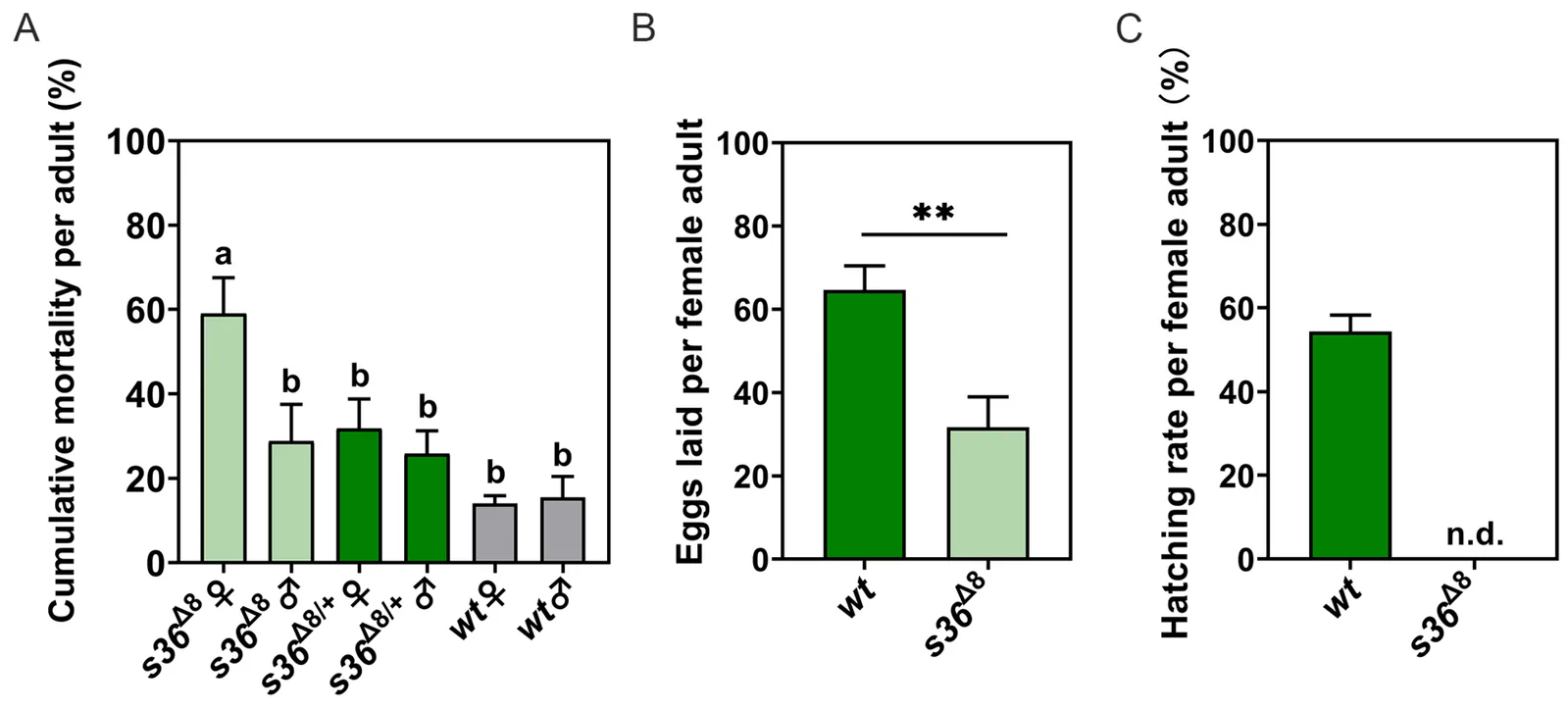
**Survival and reproductive phenotypes of *s36^Δ8^* mutants.** (**A**) Cumulative mortality per adult at 21 d after eclosion for *wt*, *s36^Δ8/+^* and *s36^Δ8^* females and males. Different lowercase letters indicate significant differences between groups by Duncan’s multiple range test (*p* < 0.05). (**B**) Number of eggs laid per female over seven consecutive days. Data are shown as mean ± SEM (*n* = 3). ** indicates significant differences at *p* < 0.01 (Student’s *t*-test). (**C**) Egg hatching rate per female. Data are shown as mean ± SEM (*n* = 3). n.d., not detected.

To determine whether disruption of *s36* affects adult survival, we compared cumulative mortality at 21 d after emergence among the four genotypes. Mortality of homozygous mutant females reached 59%, significantly exceeding that of homozygous mutant males (28.78%), heterozygous females (31.79%) and heterozygous males (25.80%), which did not differ from one another (Figure 2A). These results indicated that the survival cost of *s36* disruption is confined to homozygous females and emerges after sexual maturation.

To assess the consequences of *s36* disruption for reproductive output, we recorded oviposition and egg hatching over seven consecutive days. Wild-type females laid 64.67 eggs per female, whereas homozygous mutant females laid 31.67 eggs per female, a reduction of 51.03% (*p* < 0.01; Figure 2B). The contrast in egg viability was more pronounced. Wild-type eggs hatched at 54.34%, whereas none of the eggs laid by homozygous mutant females hatched during the observation period (Figure 2C). Because all replicate values in the mutant group were zero, this comparison was not tested statistically. These results indicated that *s36* is required both for normal egg production and more strictly for the production of viable eggs.

### 3.3. Loss of s36 Impairs Ovarian Development and Eggshell Integrity

**Figure 3:**
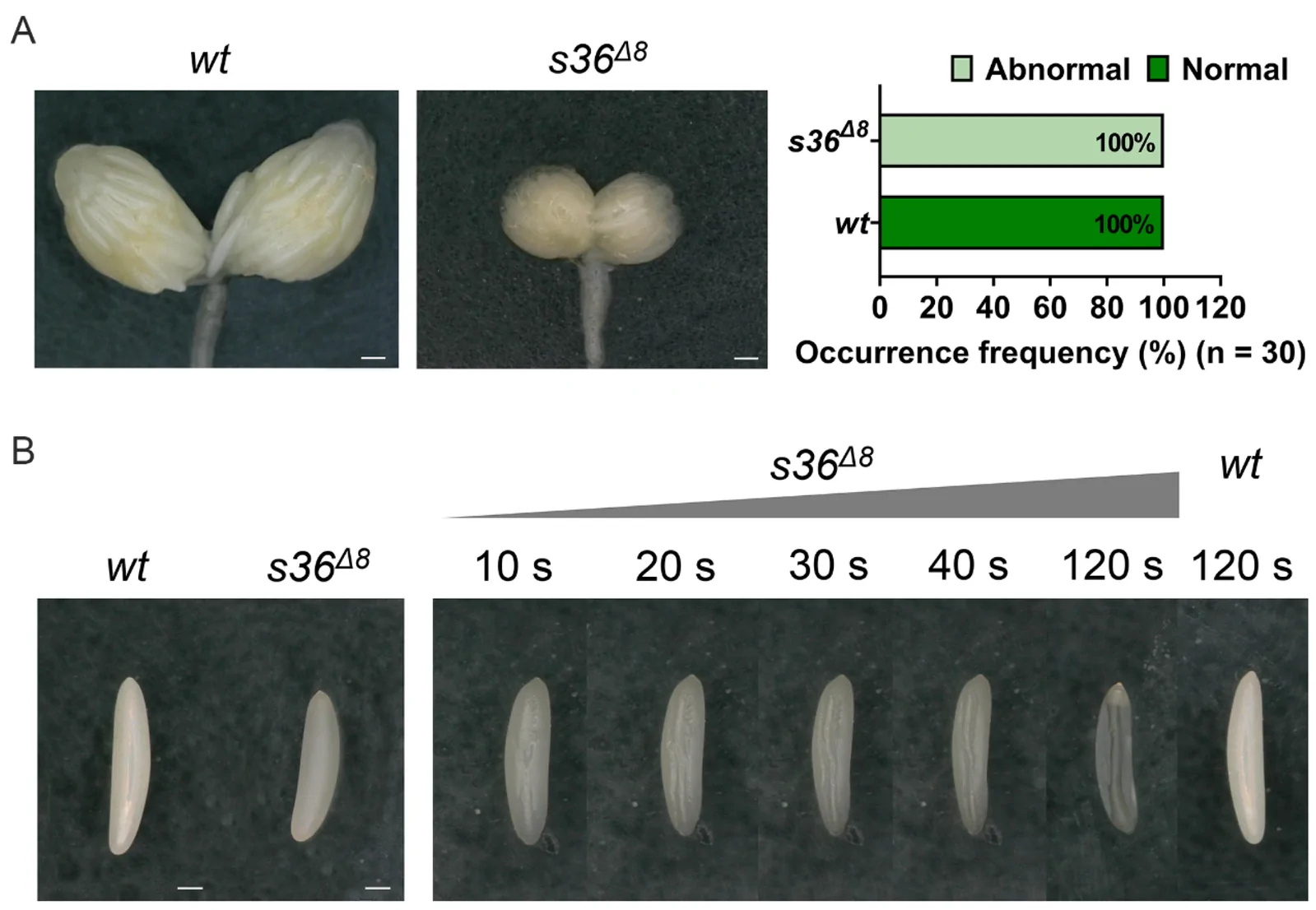
Ovarian and egg morphology of *s36^Δ8^* mutant females. (**A**) Ovaries dissected from sexually mature wild-type and homozygous mutant females. The proportion of ovaries scored as normal or abnormal (*n* = 30). Scale bar indicates 100 µm. (**B**) Morphology of eggs laid by wild-type and homozygous mutant females (left) and the progressive shriveling of mutant eggs at 10, 20, 30, 40 and 120 s after exposure to air, with a wild-type egg at 120 s shown for comparison (right). Scale bar indicates 100 µm.

To localize the defect within the reproductive tract, we dissected ovaries from sexually mature females. Ovaries of homozygous mutant females were markedly reduced in size, and no mature oocytes were visible within the ovarioles under the imaging conditions used, in contrast to the ovarioles of wild-type females, which were filled with mature oocytes. Each mutant ovary examined was scored as abnormal, whereas every wild-type ovary was scored as normal (Figure 3A). Eggs that were nevertheless laid by mutant females were smaller and duller than wild-type eggs, and their surface was rough and had lost its luster entirely (Figure 3B). When freshly laid eggs were exposed to air on a glass slide, mutant eggs shriveled progressively within 120 s, whereas wild-type eggs retained their shape over the same interval (Figure 3B). We also found that, compared with wild-type, eggs of homozygous mutants showed a significant reduction in weight with increasing exposure time in air (Figure S1). These results indicated that loss of *s36* impairs oocyte maturation and yields an eggshell that no longer retains water effectively, consistent with the complete failure of these eggs to hatch.

### 3.4. Transcriptomic Response to s36 Disruption

**Figure 4:**
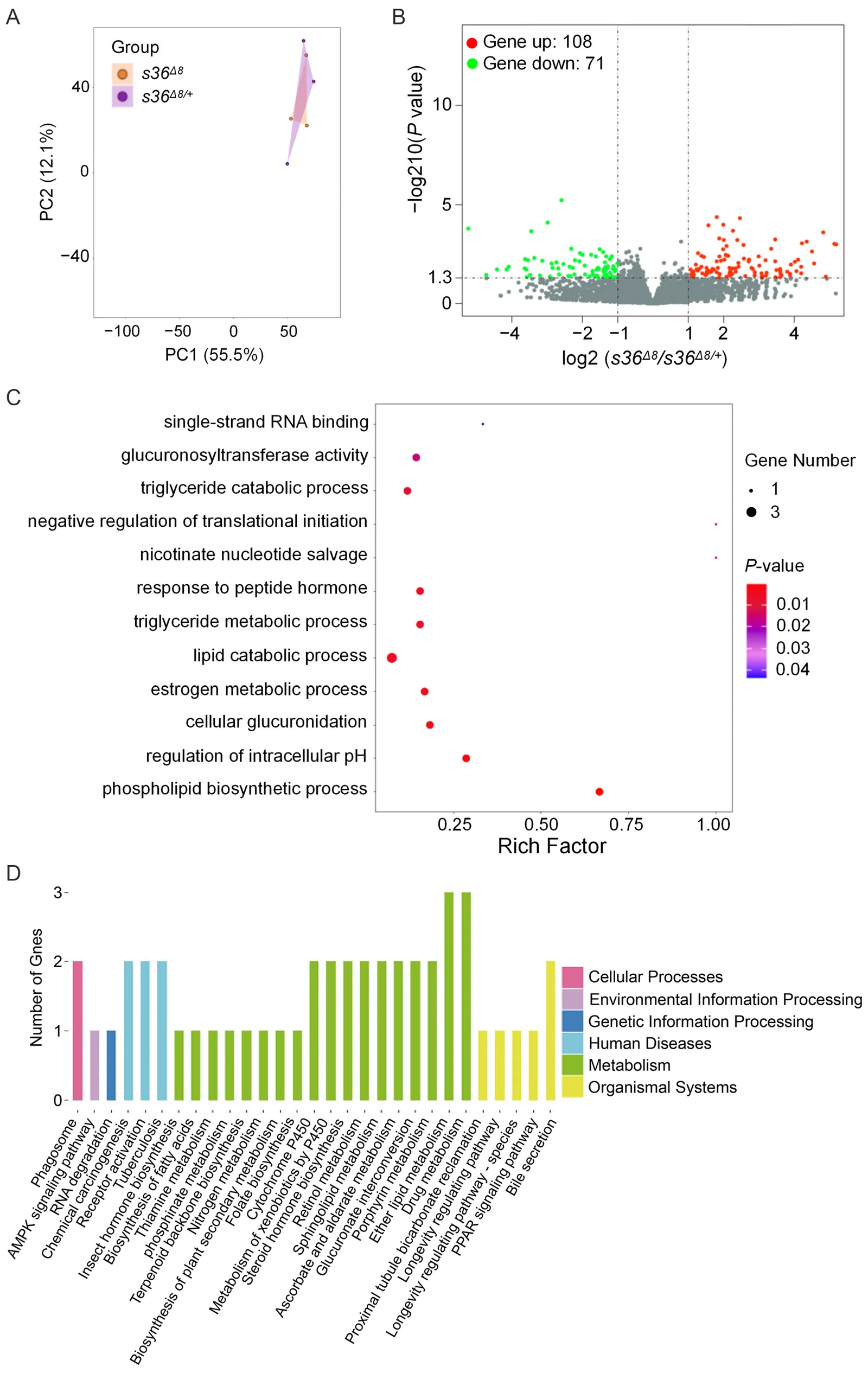
Whole-body transcriptome profiling of *s36^Δ8^* homozygous versus *s36^Δ8/+^* heterozygous females. (**A**) Principal component analysis of gene expression among three biological replicates of *s36^Δ8/+^* and *s36^Δ8^*. (**B**) Volcano plot of differentially expressed genes between *s36^Δ8/+^* and *s36^Δ8^* adults after sexual maturity. The red and green points denote significantly up- and down-regulated genes, respectively. (**C**) Gene Ontology enrichment of differentially expressed genes. Bubble size indicates the number of genes assigned to each term and color indicates the *p*-value. (**D**) KEGG pathway classification of differentially expressed genes. The bar color indicates the functional category.

To explore the molecular basis of these phenotypes, we compared the whole-body transcriptomes of homozygous mutant and heterozygous females. Heterozygotes rather than wild-type individuals were used as the reference here because the line is maintained by heterozygote intercrossing, so that homozygotes and their controls are siblings from the same cross and share genetic background. Principal component analysis separated the two genotypes along the first component, which explained 55.5% of the total variance, with the second component explaining a further 12.1% (Figure 4A). However, one heterozygous replicate lay close to the mutant cluster along PC1, indicating appreciable within-genotype variability that should be borne in mind when interpreting the differential-expression results. Differential-expression analysis of the RNA-seq libraries identified 179 DEGs, comprising 108 up-regulated and 71 down-regulated genes in homozygous mutants relative to heterozygous controls (Figure 4B). The full list of differentially expressed genes is given in Table S3. GO enrichment of these DEGs recovered terms associated with lipid turnover, including lipid catabolic process, triglyceride metabolic process, triglyceride catabolic process and phospholipid biosynthetic process, together with cellular glucuronidation, glucuronosyltransferase activity, response to peptide hormone and regulation of intracellular pH (Figure 4C). KEGG classification assigned the majority of the annotated DEGs to metabolic pathways, among them insect hormone biosynthesis, steroid hormone biosynthesis, sphingolipid metabolism, retinol metabolism and drug metabolism by cytochrome P450, with smaller numbers assigned to organismal systems, human diseases, cellular processes and information processing categories (Figure 4D). These results suggested that disruption of *s36* is accompanied by a shift in lipid and hormone-related metabolism in adult females, although the individual genes underlying this shift remain to be validated.

### 3.5. Vitellogenin and Yellow-g2 Transcripts Accumulate in the Fat Body but Are Depleted in the Ovary

**Figure 5:**
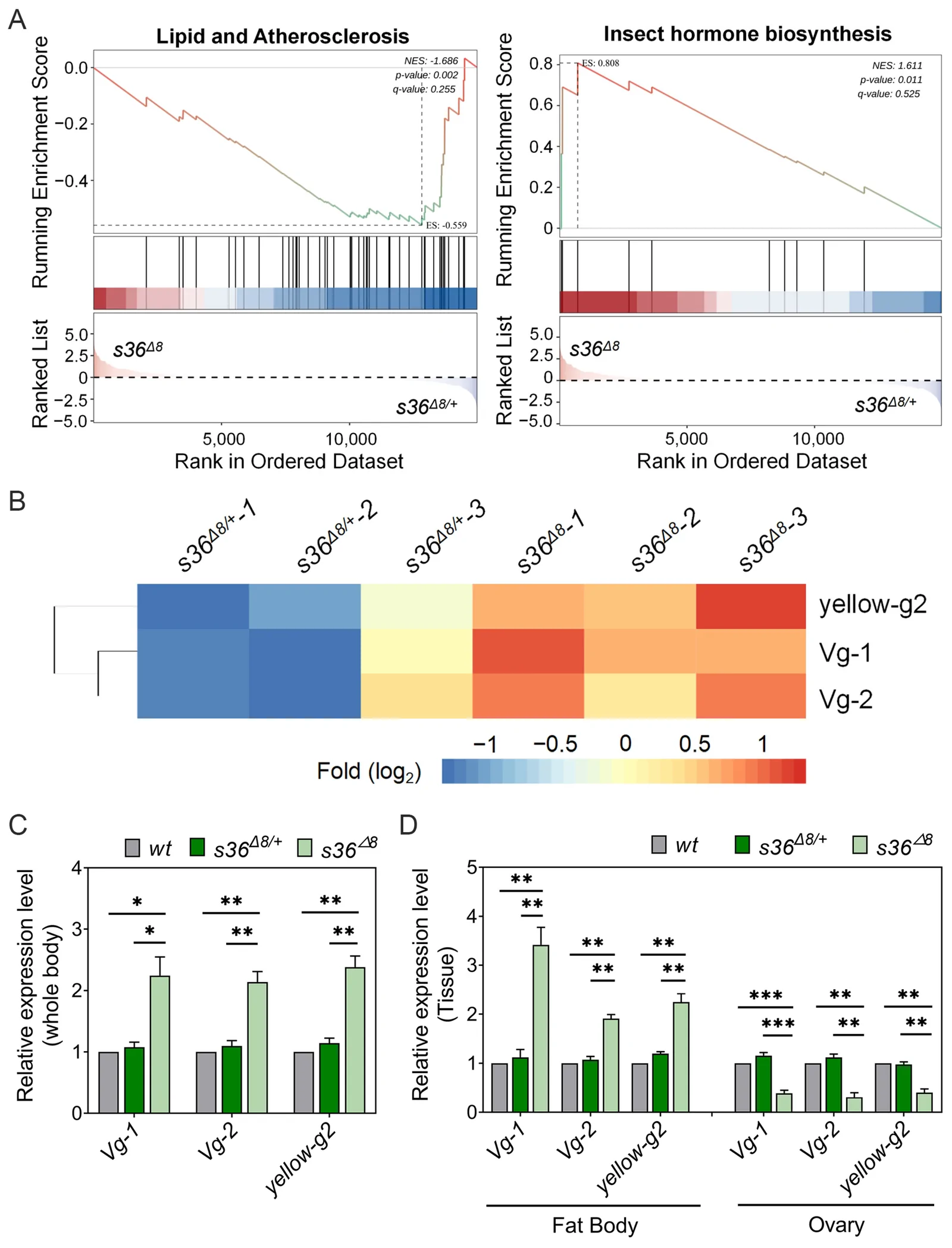
Tissue-resolved expression of vitellogenin and *yellow-g2* in *s36^Δ8^* and *s36^Δ8/+^* females. (**A**) Gene set enrichment analysis of two representative KEGG gene sets. Lipid and atherosclerosis (left), suppressed in homozygous mutants, and insect hormone biosynthesis (right), positively enriched. Normalized enrichment score (NES), nominal *p*-value and false discovery rate *q* value are shown in each panel. (**B**) Heat map of *Vg-1*, *Vg-2* and *yellow-g2* expression across the six sequenced whole-body samples, shown as log_2_ fold change. (**C**) Relative expression of *Vg-1*, *Vg-2*, and *yellow-g2* in whole bodies of *wt*, *s36^Δ8^* and *s36^Δ8/+^* females, with *rps3* as the reference gene and the *wt* level set to 1. Data are shown as mean ± SEM (*n* = 3). (**D**) Relative expression of *Vg-1*, *Vg-2* and *yellow-g2* in dissected fat bodies and ovaries, with *rps3* as the reference gene and the *wt* level set to 1. Data are shown as mean ± SEM (*n* = 3). *, **, and *** indicate significant differences at *p* < 0.05, *p* < 0.01, and *p* < 0.001, respectively (Student’s *t*-test).

Because the differentially expressed genes were few and individually of modest effect, we additionally applied gene set enrichment analysis (GSEA), which ranks all expressed genes rather than relying on a threshold-defined subset. Fourteen KEGG gene sets reached a nominal *p* < 0.05 (Table S4). None of them, however, remained significant after correction for multiple testing (lowest false discovery rate *q* = 0.255), so the enrichment results below are reported as exploratory and are not used as evidence of a specific mechanism. The three most strongly enriched were the longevity regulating pathway (NES = −1.84, *p* = 0.001), endocytosis (NES = −1.70, *p* = 0.002) and lipid and atherosclerosis (NES = −1.69, *p* = 0.002), each suppressed in homozygous mutants, whereas insect hormone biosynthesis was the only positively enriched set (NES = 1.61, *p* = 0.011; Figure 5A).

Two of these observations converged on vitellogenin. Endocytosis, the suppressed set with the clearest reproductive relevance, is the route by which vitellogenin is taken up from the hemolymph into developing oocytes [22]. At the same time the sequencing data showed that *Vg-1*, *Vg-2* and *yellow-g2* were all more abundant in whole bodies of homozygous mutants than in heterozygous controls (Figure 5B). It is worth noting that one heterozygous replicate deviated from the other two in this panel, consistent with the within-genotype variability noted above. RT-qPCR on whole bodies confirmed the up-regulation, with all three transcripts approximately twofold higher in mutants (Figure 5C). Separating the two tissues resolved the apparent contradiction: in the fat body all three transcripts were elevated in mutants, with *Vg-1* showing the largest increase, whereas in the ovary all three were reduced to roughly one quarter to two fifths of the control level (Figure 5D).

## 4. Discussion

The insect eggshell is assembled during the terminal stages of oogenesis from a defined set of chorion proteins, and its architecture determines whether the embryo is protected from mechanical damage and desiccation and whether sperm can enter the oocyte [41]. Chorion genes are among the most highly and most precisely regulated genes of the follicular epithelium, and the *s36* gene in particular shows conserved coding and regulatory features between *D. melanogaster* and *C. capitata*, indicating that its role has been maintained across dipteran lineages separated by considerable evolutionary distance [41]. In the present study, we provide direct genetic evidence that *s36* is required for female reproduction in *Z. cucurbitae*.

Here, we showed that disruption of *s36* abolished egg hatching completely while reducing the number of eggs laid by approximately half (Figure 2B,C). The eggs that were laid were small, dull and rough-surfaced, and they lost their shape within seconds of exposure to air. Previous studies have shown that targeted downregulation of s36 in *D. melanogaster* compromises chorion biogenesis and disorganizes the architecture of the eggshell [45], and that follicles depleted of s36 are unable to support sperm entry [46]. Our morphological observations are consistent with these findings and extend them to a pest. The loss of surface luster and the rapid shriveling we observed are the phenotypic signature of an eggshell that no longer forms a competent barrier. Because we did not quantify water loss or examine eggshell ultrastructure, the structural basis of this defect needs to be confirmed by scanning or transmission electron microscopy in future studies. However, the line was generated with a single sgRNA, so an off-target contribution cannot be formally excluded. The target site was selected for low predicted off-target probability, the phenotype co-segregated with the *s36^Δ8^* allele through successive generations of genotype-based selection, and it reproduces the eggshell defects reported after *s36* knockdown in *D. melanogaster* [45,46]; nevertheless, sequencing of the highest-ranked predicted off-target sites, an independent allele generated with a second sgRNA, or phenotypic rescue would be required to establish causality beyond doubt.

Notably, the reproductive deficit of *s36* mutant females was not restricted to the eggshell. Ovaries were markedly reduced in size and were scored as abnormal in every mutant ovary examined (Figure 3A), and both vitellogenin genes examined were significantly down-regulated in the ovary. *Vg* is the principal yolk precursors of insects and is taken up by the developing oocyte through a receptor-mediated pathway [21,22,23], and interference with vitellogenesis or with vitellogenin uptake reduces fecundity in a range of pests, including *Z. cucurbitae* [27,30,60,61]. One interpretation is that s36 acts only at the eggshell and that the reduction in yolk gene expression is a secondary consequence of arrested oocyte maturation, since chorion deposition and vitellogenesis are temporally coupled within the same follicle. An alternative is that loss of a major follicular secretory product perturbs the follicular epithelium more broadly and thereby feeds back on yolk uptake. Our data cannot distinguish these possibilities, and the position of s36 relative to vitellogenesis deserves further experimental validation.

Mortality after sexual maturation was elevated in homozygous mutant females but not in mutant males or in heterozygotes of either sex (Figure 2A). A sex-limited cost of this kind is expected for a gene expressed in the follicular epithelium, and it most likely reflects the physiological burden of an ovary that repeatedly initiates but cannot complete oogenesis rather than a direct requirement for s36 in somatic tissues. Comparable female-biased fitness costs have been reported when reproduction-related genes are disrupted in other pests [28,29]. We cannot exclude, however, that the accumulation of unreserved material in the mutant ovary contributes independently to the shortened lifespan.

The whole-body transcriptomic comparison between homozygous and heterozygous females recovered a modest number of DEGs that were enriched for lipid catabolism, phospholipid biosynthesis, glucuronidation and hormone-related pathways (Figure 4). Lipids are the dominant energy and structural reserve of the developing oocyte, and their supply depends on elongase-mediated fatty acid processing during ovarian development [62], while juvenile hormone and 20-hydroxyecdysone signaling coordinate the timing of vitellogenesis and follicle maturation in insects [24,25,26,63]. Ecdysone signaling also drives the terminal stages of follicle maturation that immediately precede eggshell deposition [64,65]. The enrichment we observed is therefore compatible with a female whose reproductive program is arrested and whose metabolic resources are consequently redirected, but the small number of genes involved and the absence of functional validation mean that this interpretation remains provisional.

A more specific reading is offered by the tissue-resolved expression data (Figure 5C, D). In insects, vitellogenin is synthesized principally in the fat body, released into the hemolymph and taken up by the growing oocyte through receptor-mediated endocytosis [22], although ovarian synthesis by the follicle cells themselves has also been documented in several species in *D. melanogaster* [66,67] and in *Rhodnius prolixus* [68], a route whose disruption in *Zeugodacus* and other insects impairs vitellogenin uptake and ovarian development [27,30]. In *s36* mutants the two vitellogenin genes and *yellow-g2* were more abundant in the fat body but less abundant in the ovary, and the endocytosis gene set was among those suppressed in the whole-body comparison. Yellow-g and yellow-g2 were reported to be expressed predominantly in the ovary in other insects, such as in *D. melanogaster* where both genes were amplified and expressed in the follicle cells during choriogenesis [69], and they were described as ovariole-specific in *Tribolium castaneum* [70] and ovary-specific in *Aedes albopictus* [71]. Our measurements were expressed relative to the wild-type level within each tissue rather than as absolute abundance across tissues, so the elevated fat body signal in mutants indicates a change relative to controls within that tissue and does not imply that *yellow-g2* was predominantly a fat body transcript in *Z. cucurbitae*. The simplest account consistent with these observations is that yolk precursors continue to be produced, and may even accumulate at their site of synthesis, but are not effectively delivered to or retained by the oocyte. This would also explain why ovarian vitellogenin transcript is reduced against both wild-type and heterozygous references while whole-body transcript is raised. We emphasize that this remains an inference from transcript abundance rather than a demonstrated mechanism. The enrichment did not survive false discovery correction, and vitellogenin protein in the hemolymph, ovary and oocyte was not measured. An equally plausible alternative is that the reduced ovarian transcript abundance of *Vg-1*, *Vg-2* and *yellow-g2* simply reflects the loss of the follicular tissue in which these genes are normally expressed, since mutant ovaries are markedly atrophied, with the elevated fat body signal following from the absence of a functional sink rather than from a transport defect. Because RNA was extracted from whole bodies of females that differ markedly in ovarian mass, differences in tissue composition may likewise contribute to the transcriptomic and pathway-level differences we report. Distinguishing these possibilities will require further protein quantification, localization or uptake assays.

From an applied perspective, the phenotype we describe is attractive for genetic control. Complete failure of egg hatching, rather than a partial reduction in fecundity, is the outcome that matters for population suppression, and it was achieved here by disrupting a single gene. Therefore, two translational directions will follow. First, homozygous loss of *s36* produces fully sterile females while males and heterozygotes remain viable, which is the genotype profile required by female-killing or sterile-insect approaches. Second, because the phenotype is an eggshell that no longer retains water, *s36* and its interacting chorion genes are candidate handles for increasing embryonic permeability. Delivery of dsRNA or other bioactive molecules to eggs is currently limited by the chorion barrier, and this limitation is most acute in Diptera whose eggs are laid inside host tissue. Combining chorion manipulation with the nuclease-co-silencing strategies now used to stabilize ingested dsRNA in tephritids would be a concrete next step. Genome editing is increasingly used to build self-limiting or sex-distorting strains for pest management [53,56,72], most often by targeting the sex determination cascade [73,74,75,76], and reproduction-related genes validated by knockout provide the raw material for such systems [54,55]. Targeting a structural gene of the eggshell is complementary to these strategies, because it removes egg viability without altering sex ratio or requiring sex-specific expression. Whether these advantages translate into field efficacy will depend on the fitness of released individuals and on the stability of the introduced allele, both of which remain to be established.

## 5. Conclusions

In this study, we generated a homozygous *s36* mutant line of *Z. cucurbitae* by CRISPR/Cas9 mutagenesis and characterized its reproductive phenotype. Loss of *s36* reduced transcript abundance to a small fraction of the wild-type level, halved the number of eggs laid, abolished egg hatching entirely, produced markedly atrophied ovaries that were scored as abnormal in every mutant female examined, and produced eggs that were dull in appearance and became visibly shriveled within seconds of being exposed to air. Vitellogenin genes were down-regulated in the ovaries of mutant females while being elevated in the fat body, and the whole-body transcriptome showed a shift towards lipid and hormone-related metabolism together with a nominal, exploratory suppression of endocytosis. These transcript-level observations are consistent with, but do not demonstrate, impaired delivery of yolk precursors to the oocyte, and they may equally reflect the reduced follicular tissue content of atrophied mutant ovaries. These findings establish *s36* as required for normal ovarian maturation and for egg viability in *Z. cucurbitae*, and identify a candidate target that will facilitate the development of reproduction-based genetic control of this globally invasive pest.

## Data Availability

No new data were created or analyzed in this study. Data sharing is not applicable to this article.

## Supplementary Materials

The following supporting information can be downloaded at: https://www.mdpi.com/article/10.3390/insects17101024/s1, Figure S1: The wight of ten eggs laid by wild-type and homozygous mutant females at 0, 20, 40, 60, 80, 100 and 120 s after exposure to air. Data are shown as mean ± SEM (*n* = 3). Different lowercase letters indicate significant differences between *s36^Δ8^* groups by Duncan’s multiple range test (*p* < 0.05). Table S1: Primers used in this study. Table S2: Mutation types recovered in the F1 generation after CRISPR/Cas9 targeting of *s36*. Table S3: List of differentially expressed genes between homozygous and heterozygous females. Table S4: GSEA between homozygous and heterozygous females.
