# Supplementary material for: CRISPR/Cas9-Mediated Knockout of the Chorion Gene s36 Impairs Ovarian Development and Abolishes Egg Hatching in the Melon Fly Zeugodacus cucurbitae
Source: Insects. 2026 Oct 4;17(10):1024. doi: 10.3390/insects17101024 (PMC13704921; doi:10.3390/insects17101024)
Supplement: Supplementary file 1 [file insects-17-01024-s001.zip › Table S1.pdf]

Table S1 PCR primers used in this study

Primers	Primer sequence
**For genes detection**	
Qs36-F	5'-AGCACCCGCACCATCTTATA-3'
Qs36-R	5'-AGATGCTTCAGCGCCGTAT-3'
QVg1-F	5'-GAACTGGTTGAAGCTTGCAAAC-3'
QVg1-R	5'-GTGGCACCTTGATTGTCCTT-3'
QVg2-F	5'-AATCAGTTGGTATCCACCGC-3'
QVg2-R	5'-GAAGTATCCTGCTCAGAATCATC-3'
Qyellow-F	5'-ACTTAAACCGGGCGCATGTC-3'
Qyellow-R	5'-GCCGGTGTCTAACACCCAG-3'
QZcRPS3-F	5'-TAAGTTGACCGGAGGTTTGG-3'
QZcRPS3-R	5'-TGGATCACCAGAGTGGATCA-3'
**For sgRNA synthesis and genotyping**	
sgRNA-s36-F2	5’-TAATACGACTCACTATAGGCCAGCCTGTTGAGCACC ACGTTTCAGAGCTATGCTGGAA -3’
sgRNA-R	5’-AAAAAAAGCACCGACTCGGTGCCAC-3’
gRNA-s36-F	5’-CCATCGGTGTAAACAGATCA-3’
gRNA-s36-R	5’-TTACGATAGACTGGGTTGGAG-3’

F: Forward; R: Revers
