# Supplementary material for: CRISPR/Cas9-Mediated Knockout of the Chorion Gene s36 Impairs Ovarian Development and Abolishes Egg Hatching in the Melon Fly Zeugodacus cucurbitae
Source: Insects. 2026 Oct 4;17(10):1024. doi: 10.3390/insects17101024 (PMC13704921; doi:10.3390/insects17101024)
Supplement: Supplementary file 1 [file insects-17-01024-s001.zip › Table S2.pdf]

Table S2 Mutation types recovered in the F1 generation after CRISPR/Cas9 targeting of *s36*

Genotype	Base change (△Insert ▽Delete)	Protein change (aa)	Frame Shift
$s36^{\Delta 8}$	△4 & ▽21	318→59	Yes
$s36^{\Delta 19}$	▽1	318→95	Yes
$s36^{\Delta 25}$	▽12	▽4	No
$s36^{\Delta 81}$	△1 & ▽7	△2	No
$s36^{\Delta 2427}$	△1 & ▽3	318→134	Yes
$s36^{\Delta 232}$	△1 & ▽2	318→103	Yes
